# Design Guidelines for Database-Driven Internet of Things-Enabled Dynamic Spectrum Access

**DOI:** 10.3390/s21093194

**Published:** 2021-05-04

**Authors:** Dayan A. Guimarães, Elivander J. T. Pereira, Antônio M. Alberti, Jonas V. Moreira

**Affiliations:** National Institute of Telecommunications (Inatel), S. R. Sapucaí 37540000, MG, Brazil; elivander@mtel.inatel.br (E.J.T.P.); alberti@inatel.br (A.M.A.); jonas@inatel.br (J.V.M.)

**Keywords:** blockchain, cognitive radio, distributed ledger technology, dynamic spectrum access, Internet of things, spectrum sensing

## Abstract

The radio-frequency spectrum shortage, which is primarily caused by the fixed allocation policy, is one of the main bottlenecks to the deployment of existing wireless communication networks, and to the development of new ones. The dynamic spectrum access policy is foreseen as the solution to this problem, since it allows shared spectrum usage by primary licensed and secondary unlicensed networks. In order to turn this policy into reality, the secondary network must be capable of acquiring reliable, real-time information on available bands within the service area, which can be achieved by means of spectrum sensing, spectrum occupancy databases, or a combination of them. This Review presents guidelines related to the design of a framework that can be adopted to foster dynamic spectrum access policies. The framework applies special-purpose Internet of Things (IoT) devices that perform spectrum sensing, subsequently feeding a spectrum occupancy database, which in turn will be used by the secondary network to gather information on location-dependent spectrum availability. The guidelines address technological enablers capable of making the framework feasible, reliable and secure.

## 1. Introduction

The scarcity of free bands in the radio-frequency (RF) spectrum is known to be a severe obstacle to the deployment of existing wireless communication networks, as well as to the development of new ones. The RF spectrum shortage problem is mainly owed to the current fixed allocation policy, in which the incumbent or primary user (PU) is granted exclusive access to a portion of frequencies that depends on the provided service. This problem tends to worsen due to the massive deployment of the Internet of things (IoT) and the fifth-generation (5G) of communication networks [1,2], since it is expected an unprecedented growth in the number of connections, data rates and services in these networks in comparison with current ones.

A promising solution for alleviating the RF spectrum shortage problem is the dynamic spectrum access (DSA) policy, which has being strengthened since the arrival of the cognitive radio (CR) concept [3], recognized as a candidate for being one of the key enablers of the 5G networks and beyond [2]. In DSA, shared spectrum access between PU and secondary user (SU) networks is allowed, which significantly increases the efficiency of the RF spectrum usage.

Dynamic spectrum sharing and access can be considered important requirements for future wireless networks, and as such they define the first design dimension (D1) in the present framework. This dimension, along with other ones addressed throughout this Review, are summarized in Table 1.

Among the possibilities of shared access, this Review considers the opportunistic (or interweave) strategy, in which frequency bands unoccupied by the PU network are allocated to the cognitive SUs in a non-interfering basis. An SU transmits simultaneously with a PU only in the event of a false spectral hole detection [4]. This strategy carries lower implementation complexity (D5) and higher chances of acceptance, since it can offer more protection to the primary network against interferences in comparison to underlay and overlay approaches [4]. The opportunistic spectrum access is possible due to the fact that not all the allocated frequencies are in constant use by the primary network throughout the entire coverage area. For example, it has been found in [5] that even in the most crowded broadcast television spectrum zone, about 28% of the channels were identified as vacant, with this number increasing to around 60% in less crowded regions within urban areas.

To accomplish opportunistic DSA, three main approaches may be adopted. In the first, the SUs discover about free bands by querying an spectrum occupancy database where it is stored a list of available channels. This repository is often referred to as a geolocation database (GLDB) [6] or a white-space database (WSDB) [7], the latter being the most adopted term by the Institute of Electrical and Electronic Engineers (IEEE) [8], by the Federal Communications Commission (FCC) in the United States (US), and by the Office of Communications (Ofcom) in the United Kingdom (UK) [9,10]. In the second approach, a spectrum monitoring technique known as spectrum sensing [11,12] is applied to seek for vacant bands for subsequent opportunistic access by the SUs (D2). In the third approach, as specified by the IEEE 802.22 standard [8], the spectrum sensing information coming from the sensing automation process in the customer premises equipment (CPE), as well as the channel availability map retrieved from the WSDB, can be combined by the spectrum manager in the base station (BS). The final decision upon the occupation state of the targeted band is made taking into account both information. Regardless of the employed approach, reliability and consistency of spectrum information and opportunistic decisions are required (D3).

When spectrum sensing (D2) is made without the help of any other white-space seeking technique, unreliable decisions regarding the occupation state of the sensed band may result (D3). Analogously, the sole adoption of a WSDB may not be capable of providing reliable spectrum occupancy information for DSA purposes, mainly because of low accurate processes of feeding such databases, for instance those based on propagation prediction [13], and not frequent enough database updating events (D3). On the other hand, the combination of spectrum sensing with a WSDB, as predicted by the IEEE 802.22 standard, is capable of bringing improved spectrum occupancy information (D3), but may also suffer from possibly outdated database information (D4), as well as increase the complexity (D5) and energy consumption (D6) of the SU terminal due to its dedicated spectrum sensing hardware.

In this Review we address design guidelines within a novel DSA framework in which a WSDB with high confidentiality, integrity, availability, authenticity, and immutability (D7) is fed by channel availability information coming from a supporting network of spectrum sensors not belonging to the secondary network, with high enough node density (D8) and sensing update frequency (D4) to provide a fine-grid, real-time spectrum occupancy map (D9). It is aimed that only the WSDB information is eventually used by the secondary network to drive DSA, with no spectrum sensing made by the SUs. The high density (D8) and possibly high geographic extension of a low cost IoT network (D10) is explored as the spectrum sensing supporting architecture. Some of location-aware (D9) IoT devices properly selected are equipped with an spectrum sensing module, turning them into spectrum sensor IoT (SSIoT) devices. These devices provide distributed information about the actual spectrum usage throughout wide areas (D8), so that the WSDB is often updated (D4) to be used by the SU network, or even by the IoT network itself. The hardware complexity of the SUs is not increased (D5), since the spectrum sensing task is shifted from the secondary network to the IoT-based supporting network of spectrum sensors. The main enabler of the framework is the distributed ledger technology (DLT) [14,15,16,17] (D3 and D7), which supports the creation of a spectrum market (D11) and the implementation of the control plane of the solution (D3) [18]. The framework is designed as a set of distributed services that interoperate through TCP/IP. Some services will inhabit regional data centers due to latency restrictions (D4). Others run as cloud computing components (D10). The interoperability (D13) of equipment, data, services and platforms is therefore a design concern, being addressed throughout the text.

In summary, the contributions of this work, besides the technology-related review itself, are:

- A framework for DSA consisting of a supporting IoT network of spectrum sensors that feed a WSDB, which drives the DSA functionalities enabled by the DLT.
- A set of guidelines and technological enablers to support the implementation of all planes of the framework and to demonstrate its feasibility.

It is important to highlight that this Review does not tackle an actual testbed, neither its network-level computer simulation. This is justified by the large complexity of having such a testbed or its simulation model, since the addressed solution encompasses a whole DSA framework involving full-operational primary networks, secondary networks, a supporting IoT network equipped with SSIoT devices, white-space databases and all sort of supporting technologies for interconnection, control, security and administration. Regarding the design of the SSIoT devices, no restrictions on the choice of the spectrum sensing technique has been made, meaning that any of the alternatives available in the literature could be adopted [19,20,21,22].

The remainder of this Review is organized as follows. Section 2 establishes the background and technical terminology. Section 3 explores state-of-the-art initiatives or solutions for the DSA problem. A candidate DSA framework is described in detail in Section 4, with emphasis on design guidelines grounded on the dimensions defined in Table 1, and possible technological enablers, including DLT candidates. Research challenges and open issues are addressed in Section 5. Section 6 concludes the Review, summarizing the research findings and exploring some final remarks and opportunities for further contributions.

## 2. Background

Aiming at establishing the nomenclature and revising fundamental concepts, this section provides a short background on white-space database, spectrum sensing, and database-assisted spectrum sensing.

### 2.1. White-Space Database

As already mentioned, a WSDB is one of the approaches used to support DSA. It is considered by some national regulatory authorities around the world, and is already part of standards such as the IEEE 802.22 [8], the IEEE 802.11af [23], and the IETF protocol to access white-space (PAWS) [24].

The DSA when applying a WSDB consists of the following main steps: (i) the PUs register into the database, providing their geolocation and transmission characteristics, which are used to determine the exclusion zone around the PU transmitter location by means of coverage prediction models; (ii) the SU master also registers into the database, providing all operating parameters, its location and the location of slave devices; (iii) the SU master sends a request to the WSDB, asking for a list of available channels; (iv) the WSDB answers to the request with allowed operation parameters, e.g., maximum transmit power and adjacent channel emission limits, as well as the channels that can be used by the secondary network; (v) finally, the master device enables the slave devices with the received parameters.

The main drawbacks of the WSDB-assisted spectrum sharing approach are: (i) its reliability depends on the accuracy of signal coverage prediction; (ii) it does not guarantee real-time information on spectrum usage, owed to the fact that the coverage prediction models may not reflect the actual up-to-date spectrum occupation at certain locations [13,25].

In 2014, in the US, the FCC designated ten database administrators, among which four are currently approved to provide the service, namely: Spectrum Bridge, Inc.; Iconectiv; Keybridge Global, LLC; Google, Inc.; LS Telcom, Inc. and RadioSoft, Inc. RadioSoft was the fifth database administrator approved, but it has been acquired by LS Telcom, Inc. in 2014 [9,26]. LS Telecom kept the database active. Google has ended its television white-space (TVWS) project in 2018 to prioritize a new project directed to the citizens broadband radio service (CBRS), which accounts for a broadcast service, with a spectrum portion reserved to the US Federal Government to avoid interference with the US Navy radar systems and aircraft communications [27,28].

**Figure 1:**
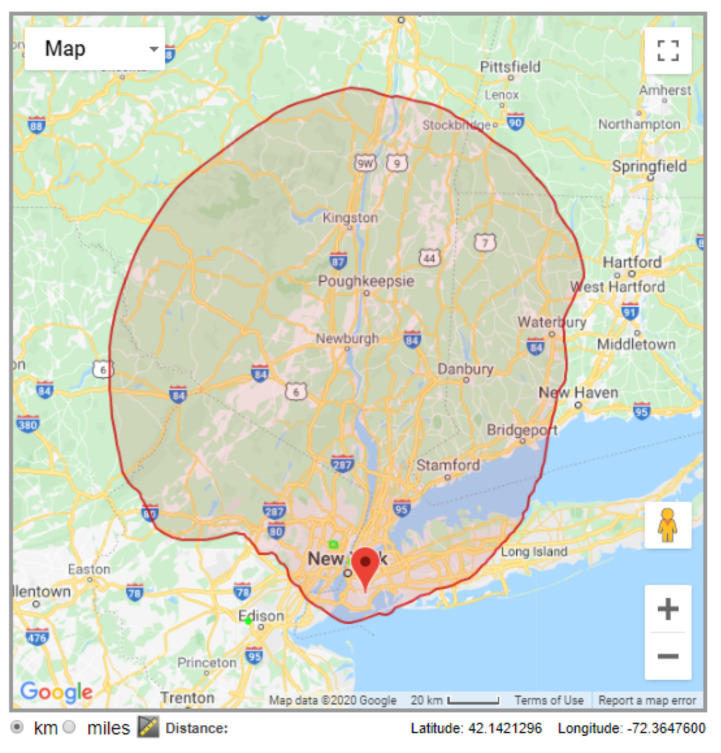
Exclusion zone for an available channel near New York city, US, as given by the LS Telcom database [29].

Using an LS Telcom database example, Figure 1 shows the exclusion zone for a vacant channel near New York city, US [29]. Since the exclusion zone is determined based on coverage prediction, it may not represent the actual coverage area of the PU signal, and may not be updated in real time. Moreover, such concept of exclusion zone does not take into account that an SU on its edge, but outside it, may cause harmful interference in a PU inside the zone. It must be also taken into consideration that interference levels from the PUs are also determined based on signal propagation predictions, thus being prone to the intrinsic inaccuracy of such methods.

**Figure 2:**
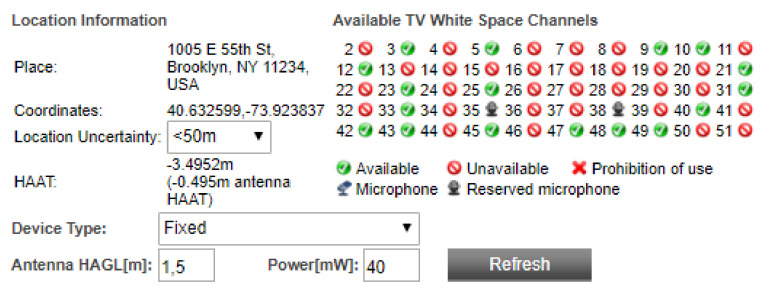
LS Telcom interface for querying channel availability. Parameters provided for a location in Brooklyn, New York, US [29].

As another example of a WSDB-assisted spectrum occupancy information, Figure 2 shows the channel availability at a given location in Brooklyn, New York city, US. This data can be retrieved from the LS Telcom database upon providing the coordinates, with a location uncertainty that must be less than 50 m, the device type, and the transmitted power and antenna height above ground level (HAGL) [29].

### 2.2. Spectrum Sensing

The spectrum sensing process can be cast as a binary hypothesis test in which the decision upon the presence or the absence of the PU signal in the sensed band is made by comparing a test statistic (also called decision variable) with a decision threshold, which is set according to the desired spectrum sensing performance. Typical test statistics are based on energy detection, matched filtering, cyclostationary feature detection, and eigenvalue-based detection [11,12]. In energy detection, which is the simplest spectrum sensing technique, the test statistic is a measure of the energy of the received signal, which differs from the absence to the presence of the PU signal. Matched filtering applies a receiving filter that is matched to the received PU signal waveform, which renders it a high implementation complexity, but optimal performance in some cases. The high complexity is also a characteristic of the cyclostationary feature detection, which explores the cyclostationarity of the PU signal to distinguish it from the noise, which is not a cyclostationary random process. Eigenvalue-based detection makes use of the eigenvalues of the received signal covariance matrix to detect the presence of the PU signal, being also complex mainly due to the need for computing the covariance matrix and their eigenvalues. There are detection techniques that have low complexity, slightly higher than the energy detection, whose test statistics are formed directly from the elements of the received signal sample covariance matrix. Among these detectors we highlight the locally most powerful invariant test (LMPIT) [20] and the Pietra–Ricci index detector (PRIDe) [22].

When spectrum sensing is made independently by each SU, unreliable decisions regarding the occupation state of the sensed band may result, mainly due to multipath fading, signal shadowing and the hidden terminal problem. Cooperative spectrum sensing (CSS) comes as an improvement, providing more reliable decisions thanks to the spatial diversity explored by SUs in different locations [11,12,22]. As the name indicates, CSS is made by a group of SUs that collaborate, jointly monitoring the spectrum occupancy.

Cooperative spectrum sensing can be centralized or distributed. The former processes the samples or local decisions forwarded by the SUs to a fusion center (FC), where the final global decision upon the occupancy state of the sensed band is made. When the samples collected by the SUs are sent to the FC, this is called a data-fusion process. When the SUs’ local decisions are transmitted to the FC, a decision-fusion process takes place. In distributed CSS, SUs in cooperation share their local decisions and the global decision is jointly reached, for instance by consensus. When a global decision is made in favor of a vacant band, it is broadcasted to the SUs to allow the subsequent opportunistic access by means of an appropriate algorithm.

**Figure 3:**
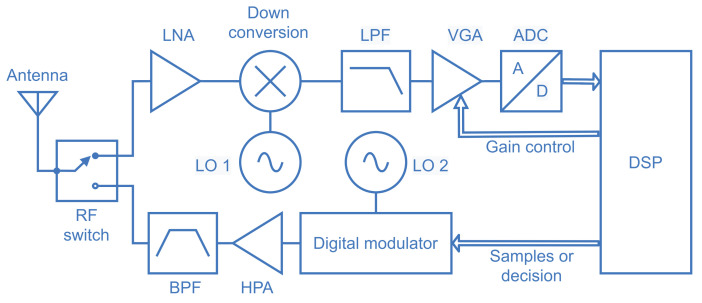
Block diagram of a receiver architecture for spectrum sensing.

To illustrate the practical aspects of spectrum sensing and show a possible internal structure of the spectrum sensing module of the SSIoT device, Figure 3 presents the diagram of a direct-conversion spectrum sensor, which is suited for software-defined radio (SDR) receivers and monolithic integration [30]. The sensed signal is captured by the antenna and directed to the low noise amplifier (LNA) through an RF switch [31]. The signal at the output of the LNA is down-converted to baseband by means of the multiplier (mixer) and the local oscillator 1 (LO 1); the LO 1 frequency determines the spectral location of the band to be sensed. The result goes through a lowpass filter (LPF) that limits the signal bandwidth and avoids aliasing. A variable gain amplifier (VGA) maintains the signal excursion within the dynamic range of the analog-to-digital converter (ADC), with the help of control bits coming from the digital signal processor (DSP). The bits representing the received signal then goes to the DSP, where the digitized received samples are just conditioned (in the case of data-fusion) or the test statistic is formed and the SU’s local decision is made (in the case of decision-fusion). The samples or decisions are sent to a digital modulator whose center frequency is determined by the LO 2, depending on the center frequency of the control channel used to communicate with the fusion center. The modulated signal goes through a high power amplifier (HPA) whose output signal is filtered by a bandpass filter (BPF). The resultant signal is then sent to the antenna via the RF switch, which is now in the opposite state. The target receiver is the fusion center.

The performance of the spectrum sensing is measured by means of the probability of false alarm, $P_{\mathrm{fa}}$, and the probability of detection, $P_{d}$. The former is the probability of declaring the PU signal present in the sensed band when the PU transmitter is in fact inactive, or the PU signal is not capable of reaching the location where the spectrum sensor is placed. The latter is the probability of declaring the presence of the PU signal when it is indeed present.

A low $P_{\mathrm{fa}}$ is desired in order to increase the chance of opportunistic spectrum access by the secondary network, increasing its data throughput. On the other hand, a high $P_{d}$ is targeted so as the primary network is protected from interference that could be caused by the secondary network when it mistakenly assumes a vacant band. As an example, the IEEE 802.22 standard establishes as requirements a maximum $P_{\mathrm{fa}}=0.1$ and a minimum $P_{d}=0.9$, with −116 dBm sensitivity to detect digital television signals [32].

It is worth emphasizing that the SU terminals, which can be as simple as sensor nodes or sophisticated as smartphones, need to be equipped with the spectrum sensing capability (D3), which adds extra complexity to the device (D5) in comparison with a transceiver-only SU, potentially increasing its size and its power consumption (D6), and possibly reducing its portability [33].

### 2.3. Database-Assisted Spectrum Sensing

To improve the reliability of the two previous approaches, the spectrum sensing can be assisted by a WSDB, forming a hybrid solution. In this case, the WSDB is explored to prevent the hidden node problem, and the spectrum sensing acts at reducing areas where coverage prediction models are not capable of reflecting the actual real-time spectrum occupation. Moreover, a database-assisted spectrum sensing can prevent harmful interference to unregistered incumbents, for example wireless microphones. As a matter of fact, in the UK, for instance, the Ofcom does not allow devices operating based on spectrum sensing only. They must comply with Ofcom white-space databases requirements. An argument from Ofcom is that sensing may not work for some channels, depending on the service provided, e.g., radio astronomy service (RAS), Earth exploration satellite service (EESS) and space research service (SRS) [10,34], due to the extremely low signal levels processed by these services.

In the hybrid approach, devices equipped with the spectrum sensing capability can retrieve the incumbent information from the WSDB and use this information to increase the spectrum sensing performance or to combine the channel occupation information from both sources to achieve a more accurate decision on the channel availability [13,33,35].

**Figure 4:**
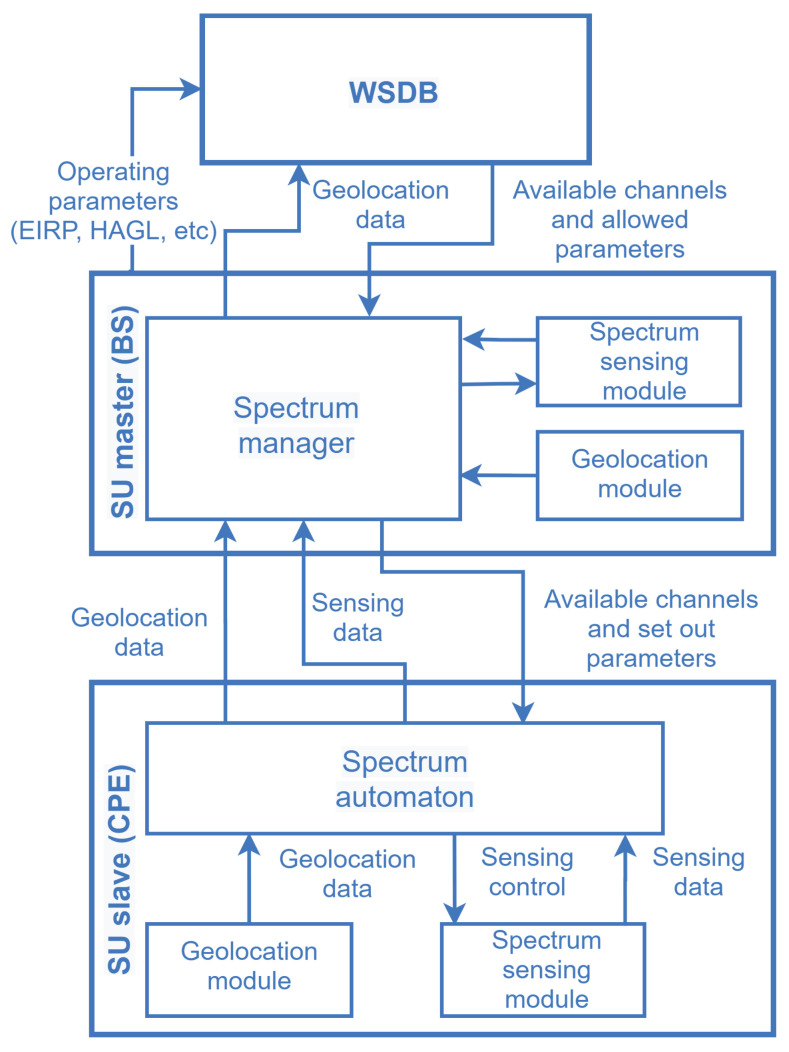
Entity relationship to coordinate spectrum sharing based on WSDB and spectrum sensing, according to the IEEE 802.22 standard.

Figure 4 illustrates a generic hybrid architecture for database-assisted spectrum sensing, from the IEEE 802.22 standard. The spectrum manager (SM), which is one of the MAC layer management entities (MLME), collects the location information and spectrum sensing reports from the end-user devices (the CPEs) and gather the geolocation-based spectrum information from the WSDB, subsequently applying policies to decide about the spectrum availability and report its decision while enabling all CPEs connected to the network. Such policies include, but are not limited to applying incumbent protection requirements from regulators and mark the channels according to some standard classification, namely: backup, protected, candidate, operating or disallowed. The SM controls the operation parameters and manages the access to spectral resources for the entire cell [36].

The main advantage of the hybrid approach is the use of both spatial and temporal white-space information [37]. However, owed to the fact that the spectrum sensing data is used in combination with the database information before making a decision on the spectrum occupancy, this data will be lost after the spectrum access and cannot be reused by other devices. The increased complexity of the SUs (D5) in order to perform spectrum sensing must be taken into account as well.

## 3. DSA-Related Solutions

This section reviews the literature concerning DSA solutions. It starts covering solutions that integrate spectrum sensing with databases, and then addresses approaches based on DLTs. A discussion on the technological gaps and research opportunities found is also carried out.

### 3.1. Integration of Spectrum Sensing and White-Space Databases

A model for building databases for television white-spaces is presented in [38], along with an algorithm to estimate incomplete data. The work addresses current methods used to estimate interferences based on channel propagation models and proposes an alternative based on spectrum sensing. Simulation results are provided to demonstrate the effectiveness of the proposed algorithm compared with current ones. The article also exemplifies the possibility of using cloud computing to improve data accuracy for a WSDB based on spectrum sensing, and algorithms to estimate incomplete measurements.

In [39], the coexistence between 5G and secondary cognitive IoT networks is explored. A transmission frame structure is proposed for DSA, in which entities are designed to collect feedback from IoT network transmissions and classify them according to spectral opportunities during the transmission of the current frame. By employing spectrum detection and quality of service (QoS) data from the channel classification, the channels with the best characteristics for transmission are prioritized for allocation in the next frame. The 5G network has primary characteristics and the IoT network is secondary. The PU is represented by any telecommunication system that holds a spectrum license, and the IoT network is idealized as a sensing infrastructure to enable DSA for any SU system.

A recent regulation concerning the CBRS has been approved by the FCC for full DSA in the 3.5 GHz radar band, also benefiting from the combination of spectrum sensing with databases [27]. The users are divided into three tiers. The higher priority users are the federal incumbents (naval radar systems and fixed satellite service) that cannot receive any interference from other users. The second level are priority access licenses (PALs), which are 10-years renewable licenses to access 10 MHz channels. PALs users are not protected from interference caused by incumbents, but they are protected from interference produced by the lower tier. The general authorized access (GAA) are license-free users who can serve to more general terminals such as fixed wireless devices, private-LTE and IoT devices. GAA users do not have any protection against harmful interference from incumbents or PALs users. PALs owners can lease their unused licenses for a determined location, or channel, or time through spectrum access systems (SASs) administrators, which is a spectrum exchange feature in CBRS. To ensure protection to the incumbents, the FCC defined as requirement the existence of a system called environmental sensing capability (ESC), made by specific spectrum sensors designated to detect naval radar signals. The ESC network feeds the SAS, a database and cloud platform designed to manage how PALs and GAA users access the spectrum. Due to the fact that these sensors are used exclusively to protect incumbents such as naval radars and fixed satellite stations, not all territory needs to be covered by the ESC network, but only regions close to the coastline and near satellite stations [28].

In [40], a review of models for DSA is presented, and a spectrum sharing scheme for 5G long-range rural networks (5G-Range) is proposed. The authors followed the CBRS regulation as a reference to classify the tiers of users for access to the spectrum. A decision structure on spectrum availability using spectrum sensing and WSDB is discussed.

The use of DSA in radar bands for cognitive radio is explored in [37]. Spectrum sensing methods are proposed to detect the pulsed radar signals, while a joint spectrum sharing system applying sensing and a location database is addressed. The database acts as an enabling center for DSA to the cognitive radio network. The authors demonstrate that the information provided by the database can assist in the sensing process of radar pulses when some data on the radar waveform is available.

A sensing infrastructure is used to create joint exclusion zones with the help of a WSDB in [41]. The authors used an open test platform located in Ljubljana, capital of Slovenia, called Log-a-Tec, which is part of the European project called the cognitive radio experimentation world (CREW) [42]. The test infrastructure has been employed to detect signals from auxiliary devices for content production, such as wireless microphones, creating a database with exclusion zones around the location where the signal was detected.

The approach studied in [43] proposes the local storage of information obtained through spectrum sensing, and the replication of data from the regulator’s database. It aims at ensuring the protection of licensed users, at the same time assisting the secondary network. The replication of WSDB data into a local database acts as a cache belonging to the secondary network, meaning that control data traffic to external networks is minimized. This locally stored data can be an entire copy, or just a part of interest of the data available on the regulator’s WSDB. The local database also assists the spectrum sensing task, acting similarly to a fusion center.

Several studies have focused on techniques to minimize the complexity of the spectrum sensing task using sampling rates below the Nyquist rate, at the same time applying a WSDB to act as an auxiliary to the detection process [13,35,44,45]. In [13], the authors adopt the strategy of storing the existing geolocation and channel propagation algorithm from the regulator’s database locally in the secondary network, similar to what is proposed in [43]. A process for the discovery of spectral holes optimized through the joint use of spectrum sensing and information received from the WSDB is discussed in [35]. These approaches are also adopted in [44,45], with different algorithms in the detection processes.

### 3.2. Distributed Ledger Technology

In [14,46,47,48], the blockchain technology has been applied to the context of DSA, where its main roles are: (i) to provide a decentralized environment for information exchange and processing, as a database, or (ii) to enable contract-based computing, storing and processing of spectrum sensing information and DSA transactions registry in a permanent and violation-proof way.

In [46], four categories of spectrum sharing based on two axes are classified. The first axis is related to primary or secondary sharing aspects and the second axis refers to cooperative or non-cooperative sharing. Primary sharing is described as the situation in which all users have equal rights to use the spectrum, similar to unlicensed access to industrial, scientific and medical (ISM) bands. Secondary sharing has hierarchical license rights, such as CBRS sharing in fixed allocation bands, as discussed in the previous subsection of this Review. Cooperative sharing allows users to interact and exchange their spectrum rights, while non-cooperative sharing is uncoordinated and the spectrum access is made opportunistically. In the special case of secondary cooperative sharing, users have access to a wider range of spectrum transactions, such as spectrum offer or lease. In this category, there is no need to have a blockchain for the whole band. Rights holders can subdivide their rights according to the arrangements they choose. This means that, for example, a primary blockchain user can manage a private blockchain with its requirements. The coordination among SUs accordingly to the established policies while avoiding interference to PUs is performed via a blockchain smart contract (A smart contract is an autonomous script that completes transactions between parts when the established requirements are satisfied. A smart contract can be used to rule the spectrum allocation process between the WSDB and the interested devices, as well as to charge fees from SUs and to pay fees to PUs, thus creating a new business model of spectrum market. The main benefit of using smart contracts is that once they are published, they are immutable and will perform exactly as they have been programmed to, with no exceptions. This characteristic can be used to guarantee the integrity of spectrum sharing operations, since they will always behave as expected). According to the authors of [46], user information that allow the identification of radio equipment or spectrum detection devices can be stored dynamically in a blockchain. However, the main function of the blockchain is to record the transactions of a spectrum market.

A service for business collaboration called full-spectrum blockchain-as-a-service (FSBaaS) is described in [47]. Two different blockchains are employed: a centralized one, called Blockchain Lite; and the Hyperledger Fabric [49], a collaborative project initiated by the Linux Foundation to support decentralized DLT. The access to both blockchains is implemented via an unified RESTful application programming interface (API), which is a kind of software architectural style for Web services such as the hypertext transfer protocol (HTTP). The centralized solution applies to customers and users that do not care about a centralizing authority that keeps track of new block registrations, whereas the decentralized option is focused on users that want to transact in a decentralized manner to maintain an independent shared ledger. The idea of using two blockchains is to take advantage of both centralized and decentralized approaches. The two blockchains share one business network manager and a common smart contract executor. The business network manager parses and manages all the business ecosystem, while the smart contract executor is responsible for the logic execution of business network solutions. The blockchain users do not maintain a blockchain node by themselves, instead trusting the tenants of the blockchain nodes that they connect to. This illustrates an important decision within a blockchain-based DSA design: which are the nodes that validate perennial records and where they will run.

In [14], a dynamic spectrum sharing solution via smart contracts over the Ethereum Blockchain is presented. A novel spectrum market digital asset called Spectral Token has been developed. This token is used to validate and track usage of licensed frequency bands. The solution enforce sequential access to spectrum by SUs in order to avoid interference. The token avoids collision and guarantees that the PUs will receive a payment for the leased spectrum. The platform allows users to get ownership of frequency bands by paying to the authority, for example regulatory agencies such as FCC. Each frequency band is coded as a spectral token and a PU may lease it to an SU. The PU may announce the lease opportunity, as well as the SU may look for free frequency bands. After defined the initiator, location, time frame, and an access fee, a certain frequency band can be negotiated through the platform. The authors of [14] also developed a proof of concept solution in the 2.4 and 5 GHz ISM bands. The performance analysis showed that the system has throughput and latency characteristics suitable to implement CBRS, IEEE 802.22 WRAN, or Small-Cell-as-a-Service use cases. However, this proof of concept does not implement all the framework requirements and capabilities described herein. It only demonstrates that it is feasible to adopt a blockchain in this sort of solution. No further details regarding transaction information and blockchain blocks are presented.

The use of smart contracts over the Ethereum Blockchain for radio-frequency spectrum market is explored in [50]. The proposal is entitled spectrum sensing as a service (Spass), in which spectrum sensing is offered as a service monetized through smart contracts. The authors also developed an algorithm for detecting malicious participants that provide false sensing information, including them in a blacklist and excluding them from future contracts.

A blockchain-empowered framework for 6G radio-frequency spectrum and infrastructure sharing is proposed in [51]. According to the authors, tokenization of wireless resources (D11) can be achieved by means of a distributed ledger structure with proper consensus algorithms and intelligent/dynamic trading. The paper considers three different types of smart contracts to determine throughput per price gains when automated spectrum trading is performed at software level. The authors contended that interference problems can be mitigated by tuning smart contracts. The framework also covers infrastructure sharing and network slicing. A comparison of consensus algorithms has been also provided to guide implementation.

In [52], the relation between blockchain and machine learning is explored. The authors argued that network operation and management would largely benefit from the cohesive integration of these ingredients (D7 and D12). Blockchains favor decentralized, secure and private machine learning training and sharing. On the other hand, machine learning can help on optimizing blockchain networks, e.g., the difficulty level on chain block validation, tuning of consensus algorithms and smart contracts, and selection of transactions to validate in a block. The authors presented a survey of techniques for blockchain and machine learning integration. Since DSA can be seen as a particular case of network operation and management problem, the integration of these technological pillars is interesting for the emergence of a radio-frequency spectrum market (D11).

The potential of blockchains for IoT is investigated in [53]. The limitations of traditional centralized IoT models are discussed, as well as the benefits and challenges behind blockchain-based IoT solutions. The authors surveyed the integration of blockchain with IoT, trustless architectures for IoT, decentralized consensus algorithms, privacy and security concerns, IoT identity management through blockchains, and monetization of IoT data. All these topics are related to the integration of IoT and databases for DSA. In summary, the authors asserted that blockchains can provide a decentralized fabric for IoT, without the need for intermediaries, fostering a dynamic IoT data and resources market.

### 3.3. Literature Gaps and Research Opportunities

Table 2 summarizes the scopes of state-of-the-art DSA solutions proposed in the literature, regarding the 12 design dimensions (D1–D12) covered in this Review. As can be seen, dynamic spectrum sharing and access policies (D1) are not addressed by several articles covering other key aspects of DSA. The few works that cover spectrum market (D11) do not take into account several other relevant dimensions for cognitive radio, such as D3–D6. This indicates that integrating the two trends covered by D11 and D3–D6 constitutes an important feature. Only a small part of the reviewed articles (33%) deals with the cost-effectiveness (D10) of the framework considered in this Review, which is a key point for most applications. This is one of the important contributions of our framework, which is the use of cheap IoT resources to provide collaborative spectrum sensing (D3) with good coverage (D8). Simplicity (D5) and energy cost (D6) are other dimensions not covered by the majority of the works. Another fundamental issue is security and immutability (D7). Most of the referred works that are concerned with D7 involve DLT, but still lack on support for D3–D6 and D8–D10 (a noteworthy exception is [50]). The framework brought by this Review covers not only the traditional security, but also the availability and immutability provided by DLTs. In short, the future of wireless networks requires solutions that address all the above-mentioned dimensions. This Review contributes to the design and specification of such integrating frameworks.

The above literature gaps and research opportunities are further explored in Section 5, where challenges and open issues are addressed.

## 4. Database-Driven IoT-Enabled DSA Framework

In this section, a novel framework aiming at driving the dynamic spectrum access policy is described. First, an overview of the solution is provided, followed by a discussion on the enabling technologies of the network-level components. Then, the WSDB design is addressed, subsequently complemented by the associated enabling technologies.

### 4.1. Overview

**Figure 5:**
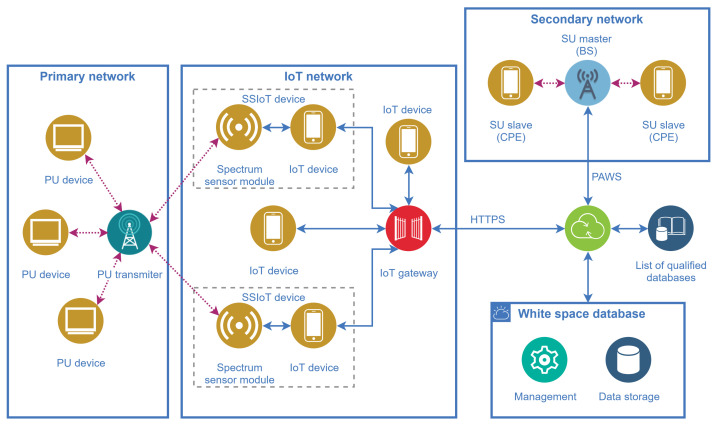
Main entities and their relationships in the database-driven IoT-enabled dynamic spectrum access framework.

The entities comprising the database-driven IoT-enabled dynamic spectrum access framework and their relationships are presented in Figure 5. All networks considered in this figure are assumed to be deployed in the same region, meaning that they have coverages in common.

**Figure 6:**
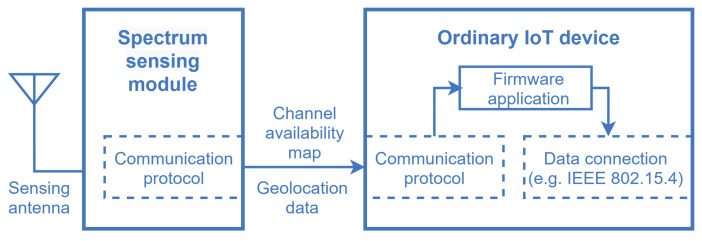
Model of an SSIoT device formed by the connection of an ordinary IoT device and an spectrum sensing module.

One of the main features of the framework is that the spectrum sensing task conventionally carried out by the SUs is shifted to SSIoT devices that are part of a supporting IoT network, reducing the complexity of the SUs. The SSIoT devices are formed by the connection of an ordinary IoT device with a spectrum sensor (SS) module, via a standard hardwired or wireless interface such as UART, I^2^C, or SPI [54], as depicted in Figure 6. The SS module sends the spectrum sensing information to the attached IoT device, along with the geolocation data if the IoT device is not equipped with a positioning feature. Subsequently, the main application layer catches the spectrum sensing information and forwards it to the gateway, and then to the WSDB.

As already mentioned, any spectrum sensing technique can be adopted in the design of the SS module, meaning that any of the alternatives available in the literature could be applied, each one carrying its pros and cons in terms of complexity and performance (see Section 2.2; see also, for instance, [11,12,19,20,21,22] and references therein).

Not all IoT devices need to be equipped with an SS module, just the quantity enough to yield the required reliability and spatial-temporal resolution of the RF spectrum occupation map that will be obtained from the spectrum measurements. In the case of wireless IoT nodes, the IoT device and the SS module may have their own antennas, since their characteristics (mainly the bandwidth and the operating frequency) may significantly differ in practice.

The high node density of the IoT network is capable of providing exclusion zones very precisely, using the spectrum sensing information from many IoT devices instead of path-loss-based exclusion zones such as the one considered in [41].

The spectrum occupancy data gathered by the IoT network and stored in the WSDB will be available to be retrieved by secondary networks interested in the dynamic access to the spectrum. The access to vacant bands is made by means of a constant monitoring of the WSDB, which can be easily accomplished during the regular control communication between the SUs and their base stations. Any SU device, equipped or not with spectrum sensing, will be able to access the spatial-temporal information about channel availability.

It is worth highlighting that the spectrum sensing information is intended to be used solely for updating the WSDB, enabling the DSA of the secondary network terminals. In other words, the DSA by the secondary network is intended to be made based only on the spectrum availability obtained from the WSDB, meaning that the SUs do not need to be equipped with the spectrum sensing feature. Nevertheless, the SSIoT devices can also be used to monitor the spectrum used by the IoT network itself, aiming at helping its nodes to access bands that are less crowded or free from strong interference.

### 4.2. Enabling Technologies—Network Level

The main application run by the SSIoT devices catches the information from the spectrum sensors and forwards it to the gateway using its particular communication protocol, e.g., compliant with the IEEE802.15.4, the Ethernet or the LoRa specifications.

The gateway of the IoT network can be a specialized node that will publish any kind of data on the Internet. The most popular protocol used in IoT devices to publish or subscribe on the Internet is the message queuing telemetry transport (MQTT) protocol. Since it is developed to resource-constrained devices, it has limitations regarding data security, for example no data encryption is provided [55]. As shown in [41,55], the security in the connection between the IoT network and WSDB can be made, for instance, with secure sockets layer (SSL) certificate, or with its successor transport layer security (TLS) certificate, using authentication via the hypertext transfer protocol secure (HTTPS) [41], keeping the network protected against malicious attacks. In this case, public and trusted certificate authorities (CA) are used by the SSL/TLS to issue digital signed certificates when authenticating clients. In [55], these mechanisms have been employed for access control security; the security of the present framework can be implemented in the same way.

Before connecting to a WSDB, the secondary network base station (the SU master BS depicted in Figure 5) may scan a list of available qualified databases in order to choose the most appropriate, according to the geographical area covered by the databases and the location of the SU terminals. The concept of qualified databases is already in use, for example, by the UK operators [56] and can be equally applied to the present framework.

After connecting to a WSDB, the secondary BS starts the registration of its network. Any SU terminal, for example a CPE in the context of the IEEE 802.22 standard, sends its geolocation information and operation parameters to the BS of the secondary network via control channels. The BS completes the registration, providing to the WSDB all required data about each SU.

According to the FCC regulations, during the registration the BS must inform the FCC identifier, the SU manufacturer and serial number, device’s antenna height above ground level and details about the device’s owner or responsible person, such as name, address, e-mail and phone number. The BS must repeat this process every time it changes any operating parameter or when an SU changes its location in a determined amount, for example when moving 100 m from where it was during the previous registry. This process and all related tasks are made by high level layers on the BS, for example the SM entity if the IEEE 802.22 is considered.

After device registration, the BS queries the WSDB about the spectrum availability for that location and receives from the WSDB the updated spectrum occupancy data, which will subsequently enable the SUs terminals to transmit in the available channel according to the parameters established by the BS.

An entity that may be added to the architecture shown in Figure 5 to extend usage scenarios is an IoT network aggregator. Its main role would be to combine data from different sources and help with the security of the network, removing more complex protocols from the resource-constrained IoT devices. A smartphone can be used to exemplify this role: when connected to smartwatches or other wearable devices that collect many user data, such as health monitoring information, the smartphone acts as a data aggregator. It removes complexity from the sensors, becoming responsible for data security and for sending all information to the gateway that will forward them to cloud servers. When the aggregator is used, it may take the role of the gateway node. In the practical model of the access control security system proposed in [55], an aggregator has been implemented with a Raspberry Pi 3, while the IoT device node has been implemented with an ESP32-DevKitC.

The refresh rate of the WSDB depends on how the spectrum sensing task is implemented. For example, this rate can be defined by the frequency in which SSIoT devices report the sensing information, but the sensor query and data dissemination protocol (SQDDP) can be used to collect the spectrum data when it is needed. The first approach spends more energy, but tends to keep the WSDB always up-to-date, whereas the second can yield a better energy efficiency, but can produce an intolerable latency to the WSDB updating process. The WSDB refresh rate may also depend on the frequency that the secondary network will access the WSDB. This might happen at least once a day for fixed devices according to FCC rules, but it could be even smaller than seconds for mobile SU devices [7].

**Figure 7:**
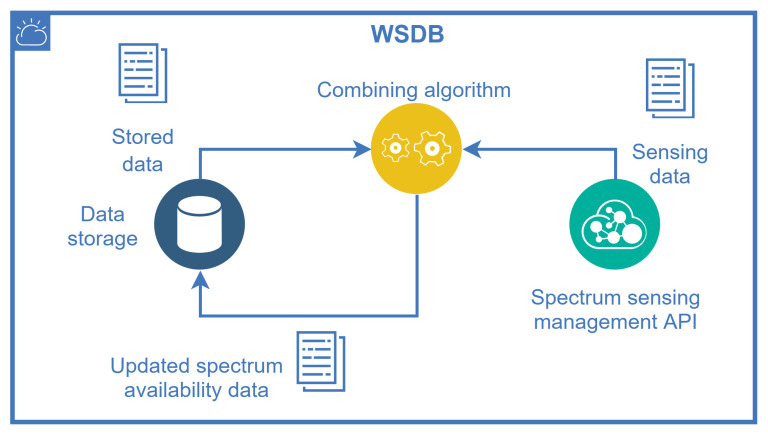
WSDB updating using hybrid sources of spectrum availability: the WSDB data itself and the spectrum sensing information coming from SSIoT devices.

The SQDDP is an application protocol proposed to sensor networks, described in [57] as a solution that provides user applications with interfaces to issue queries, respond to queries, and to collect incoming replies. These queries are not addressed to specific nodes in general, but they can be addressed to clusters at specified locations. This approach might be useful to collect real-time spectrum occupancy information “on-demand” directly from several SSIoT devices, allowing the processing of this information similarly to a cooperative spectrum sensing. A management entity, which might be part of the database, uses an algorithm to combine the geolocation information and previous stored data with the received spectrum sensing information to update its own data on channel availability. This feature is depicted in Figure 7.

Cooperation among several SSIoT devices may also be accomplished similarly to the solution proposed in [21], where a resource-shared and scalable architecture of cooperative spectrum sensors collectively processes the received signals from a number of individual sensors to improve the reliability in the detection of the spectrum occupancy.

It is worth emphasizing that the delay between the realization of the spectrum sensing and the moment that this information is made available to the WSDB depends on the network topology adopted by the IoT network and how data aggregation is made. Spectrum sensing data can be disclosed only to the closest devices or it can be sent in broadcast to all devices in the network until it arrives at the gateway, which clearly affects the latency of the information provided. According to [7], in Ofcom rules a WSDB must answer a query within 10 s. This time includes the WSDB queries to the spectrum sensors, reception of spectrum sensing information, processing of available channel map and response to the SUs. However, this answer may be needed within a much shorter time frame in the case of mobile SUs or primary networks with fast channel use update.

### 4.3. WSDB Architecture

**Figure 8:**
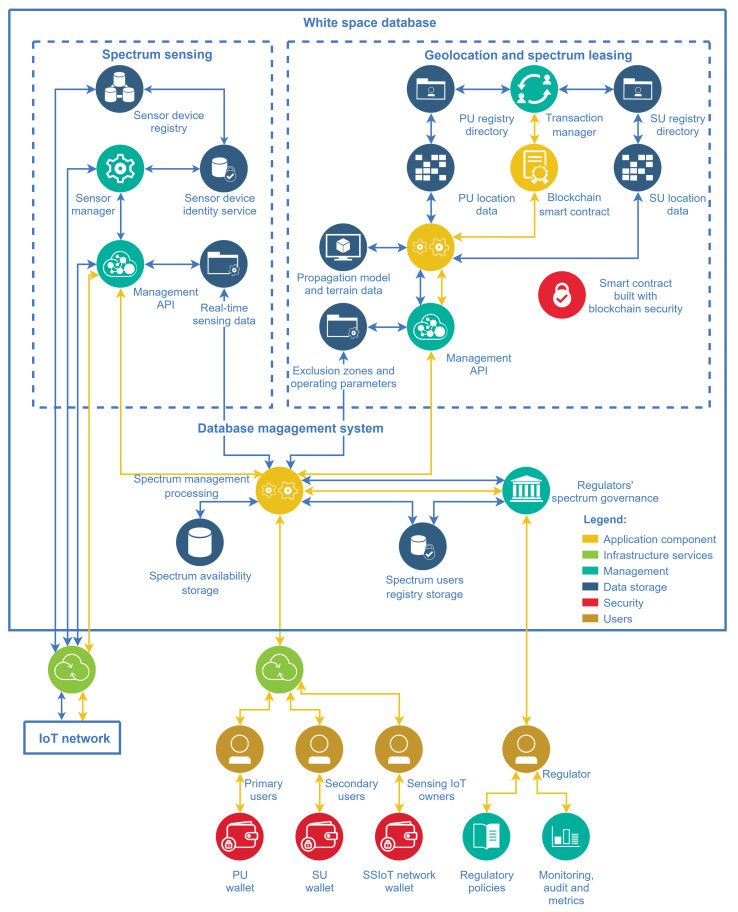
WSDB architecture with entities relationships for subsystems integration and user access.

There is not a unique way of developing a white-space database aimed at managing the dynamic spectrum access. This section focuses on presenting the authors’ view of an architecture for this task, which is depicted in Figure 8. The spectrum sensing subsystem, and the geolocation and spectrum leasing subsystem constitute the main entities of the WSDB. The components of these subsystems are described in the sequel.

The sensor device registry is the data bank that stores registries of the supporting IoT network and its SSIoT devices. The sensor device identity and localization service is responsible for the specific identification of SSIoT devices and their location, allowing the WSDB to address queries for determining clusters to perform spectrum sensing. The sensor manager refers to all functions and tools for control and communication with the SSIoT devices. The management API of the sensing subsystem comprises all features to process the spectrum sensing data, making global decisions regarding the spectrum occupation state, responding to the database’s core requests, and providing data to sensing network monetizing. The blue connections carry the data traffic with the real-time periodic sensing data forwarded to the database’s core, while the yellow connections carry the application and control flow.

The geolocation and spectrum leasing features are connected as a single subsystem in the architecture of the present framework. Composing this subsystem, the PU/SU registry directory refers to the repository where the registry and transmission settings information of the spectrum users are stored. The data from these directories are used to calculate the protection zones for the users, which are also zones where the established contracts are valid. The propagation model and terrain data refer to the necessary information to calculate these protection zones, using well-known signal coverage prediction models. The geolocation exclusion zones processing is the cloud computing facility, which calculates the protection zones based on data from users’ transmission settings, terrain data, and transaction registries from contracts. The transaction manager is responsible for applying rules to the spectrum leasing according to regulators’ policies, making possible the spectrum exchange through blockchain smart contracts. The management API, which would appear redundant in a conventional (non-DLT-based) database architecture approach, here it represents the features and functions to manage the corresponding subsystem. It provides the interface to the database’s core and is responsible for delivering the spectrum availability, the allowed operating parameters, and the protection zones.

The reason for the existence of a management API to communicate with the core in each subsystem is the different requirements that the subsystems are subjected to. Moreover, it is possible to build the two subsystems using different technologies, for example using conventional databases or employing DLTs.

Likewise, the primary users, the secondary users paying for spectrum licenses must receive protection according to the license they buy. Hence, to calculate the SUs protection zones, their contracted rights must be considered. The connection between the geolocation and the spectrum leasing features in the present architecture is also for this reason.

The database’s core composition has a storage for spectrum users registry and another one for spectrum availability data. The PU/SU registry directory previously mentioned is part of the spectrum users registry storage, whereas the spectrum data storage contains the spectrum use and channel availability. The spectrum management and processing is the cloud computing entity responsible for subsystems integration. Its roles include user registration and updated spectrum availability information delivery, issue of requests to the subsystems APIs, also acting as the interface with the regulators and providing tools to monetize users in the process of spectrum leasing. It also includes functions to implement rules, and to monitor, audit, and establish metrics to assess the effectiveness of the DSA.

### 4.4. Enabling Technologies—WSDB

The blockchain technology, when applied as a WSDB, can be exclusively employed to store the information that will be used by other components of the DSA solution, such as channel availability, spectrum sensing decisions, or even payments related to spectrum rental. Nonetheless, its use may go beyond information storage, running computer programs to process DSA information directly from the blockchain. In this case, the entire solution runs from a blockchain, being therefore immutable in terms of information storing, processing or both.

There are some implementation aspects that need to be addressed for the deployment of a blockchain-based WSDB: (i) it is necessary to decide the kind of consensus mechanism to be used when validating transactions; (ii) the cost of transactions must be taken into account, since a transaction, for example making or receiving a spectrum rental payment, has two kinds of cost to be observed: the blockchain cost, which is related with the token to be used as the payment method, and the energetic cost, which is associated with the energy used in the transaction consensus; (iii) the choice between public versus private blockchain design: a public or open blockchain is a network that anyone can join, with full participation (read, write) permissions. On the other hand, a private blockchain has restrictions on who can participate in the network; and (iv) the use of a centralized versus decentralized blockchain: a centralized blockchain has a central authority that keeps all blockchain records, while a decentralized blockchain has its data distributed over the network, without a central node to coordinate its operation.

If the DSA is meant to use a single database, it might expose the system to a weakness usually referred to as single point of failure. To handle this problem, a distributed database is more advantageous. The realization of this distributed solution requires a supporting system that implements confidentiality, integrity, availability, and authenticity. To accomplish these requirements, conventional databases such as SQL, NoSQL or both can be explored, or a DLT-based solution, or even a hybridization of these.

A database grounded on the DLT has a natural defense against the single point of failure problem due to DLT’s decentralized operation. Additionally, a consensus among all participants is necessary in order to record anything new in this database. The consensus also handles an important issue regarding distributed databases: the impossibility of storing incoherent data or duplicated entries (a duplicated entry in a DLT means a double expense to the client). This results in the guarantee that all nodes have the same data.

As the information is stored in the blockchain distributed nodes, the solution can adjust itself to deal with missing nodes. In addition, the advantage of immutable information recording makes the blockchain registers easily auditable. Any regulation body can verify the information in the blockchain as a trusted source, depending on the trust-ability of the participants who read and write from and in the blockchain, as well as on the number of nodes that validate transactions. Digital signed certificates, public cryptography and trust mechanisms can be adopted to authenticate and authorize access to the WSDB.

The use of the DLT can also improve the network security with respect to malicious devices aiming at providing incorrect spectrum occupancy data or spectrum misuse. These malicious devices could be included in a blacklist and denied from participation in future contracts.

Implementing a WSDB solution with conventional databases requires a complex infrastructure that offers security, availability, redundancy, and an adequate number of nodes to handle the network data. Achieving these requisites is costly and prone to a very time-consuming development process. An alternative is to rent some cloud service that already has traditional distributed databases or DLT implementations, depending on the selected WSDB technology. This option reduces the development time, since the responsibility about the infrastructure is of the cloud owner. Nonetheless, this option is still costly and demands the knowledge of the cloud being used.

One important advantage of public DLT-based solutions is that the required computational and networking infrastructure is shared among participating nodes, typically running in special-purpose hardware or local servers. Therefore, there is a large number of nodes globally distributed. Although possible, cloud-based solutions are not the mainstream. Independently of the DLT approach, there is always the cost of information overspread, for example the spectrum occupation and related transactions in the context of DLT-based DSA solution, to the network nodes involved in the immutable information validation and distributed storage. The ability to self-join and keep the network working properly is what it is expected from participating nodes, helping in consensus establishment and perennial data storage. This demand is shared among all network nodes, with the advantage that if one node is down, the network properties are preserved by other nodes, reducing the need for costly dedicated infrastructure. In the public approach, all the spectrum market participants access a global DLT network, relying on a general purpose solution. A DLT-based solution can also be maintained by a private consortia of stakeholders. In this case, each participant covers its own costs to run one or more DLT nodes, contributing to the final solution resiliency, as well as to the immutability of information storage and processing.

A common characteristic among existing DLTs such as Bitcoin [58], Ethereum [59] and IOTA [60] is the use of some kind of virtual currency for the transactions. For instance, the Ethereum’s coin is called *Ether* and the IOTA’s coin is the *Miota*. In order to perform any transaction in the Ethereum network, for example making or receiving a payment or exchanging data, it is necessary to have a wallet with the respective coin. Therefore, the requirement of having a wallet with coins to perform a transaction is a relevant aspect once it avoids malicious behavior. Thus, a malicious operator must get a wallet with some token before trying its threads, increasing the work to be done in order to execute some fraudulent transaction. In contrast, the IOTA allows the transfer of zero-value transactions. However, to complete a transaction, customers need to validate two previous ones, by performing a proof of work. Attackers are also unmotivated by the extra work they must do. These aspects need to be put on the scale when choosing a DLT or a combination of technologies for any DSA framework, especially if a market with payments for spectrum usage is to be deployed. In the Ethereum environment it is also possible to implement smart contracts that monetize the spectrum allocation and the IoT network that provides the spectrum occupation information, creating a new kind of spectrum market, for example as explained in [61,62].

The solution from IOTA foundation is another type of DLT called Tangle, which is a directed acyclic graph (DAG) [63,64]. The IOTA solution was motivated by solving two disadvantages of blockchain-based solutions: the high energy footprint to run a network and the transaction costs, which could be prohibitive for some applications. In contrast to the Ethereum’s blockchain, there are no miners or fees in the IOTA’s Tangle. Although the blockchain becomes slower and less productive as it grows, i.e., the time to accomplish a transaction increases, the Tangle is more scalable and keeps performance while growing. The main disadvantage of using the IOTA’s Tangle is that it does not support smart contracts as claimed in [65]. Nonetheless, the IOTA created the quorum-based computations (Qubic) protocol [66,67], which brings the smart contract functionality to the Tangle. Due to the low energy cost of transactions, IOTA is being applied to monetize IoT devices, creating what is being called a things data market [68,69]. A DSA with IOTA could therefore monetize the supporting IoT network by their work regarding spectrum sensing via SSIoT devices.

In [70], an IOTA-based control of smart city IoT devices has been explored. The paper presents a set of DLT applications for smart cities, including electrical vehicle recharging, and charging hubs for electric bikes. A solution for IoT resource sharing via dynamic deposits in an IOTA wallet has been proposed. Blockchain is compared to Tangle and a model for stability of conflicts is presented and evaluated using Monte Carlo analysis. This model can be applied for social compliance while using shared resource in a smart city. Although applied to IoT resources in smart cities, the discussion does apply to spectrum sharing in a general sense.

A review of IOTA and its potential for IoT solutions is made in [64]. A systematic review of IOTA has been provided, pointing various applications, benefits, challenges and limitations. The panorama of applications included IoT, machine-to-machine (M2M) communication, e-health, automotive industry and smart cities. The paper also presents several possible network architectures to deliver IoT sensor data to services via IOTA Tangle. Such a discussion is valid for spectral sensors and their data market, an aspect also addressed in this Review (D12).

## 5. Challenges and Open Issues

Based on the design dimensions defined in Section 1, on the research gaps and opportunities identified in Section 3.3, as well as on the technological aspects of the DSA framework discussed throughout this Review, this section explores challenges and open issues related to the framework.

### 5.1. Security and Privacy

Traditional security challenges apply to equipment, operating systems and software. In addition, there are issues related to information security before storage in the database or DLT. Threads in the physical and link layers include jamming, spectrum sensing data falsification (SSDF), control channel tampering, beacons falsification, MAC backoff window tampering, among many others. Denial of service attacks are common on the Internet and can compromise data storage and opportunistic spectrum buying and selling (D11). In DLTs, there are also double spend attacks, unwanted forks, sybil and byzantine attacks to prevent consensus (D5). Malicious contracts have already done great damage to investors in digital assets. DLTs undergo frequent updates, which require the attention of operators and users to prevent security breaches. The same is true for digital wallets. Privacy is also a concern, as not all information is public, and may compromise primary and secondary users.

### 5.2. Cloud Computing and Functions Placement

Cloud computing can be used to accommodate much of the software components. However, some may be on the edge of the regional network or local data centers, due to latency requirements (D3 and D4). In this context, an important issue is the geographic positioning (D9) of the framework’s components. Moreover, the best model for computational resources deployment is another point of concern. A public cloud has advantages, but requires adequate contracting with suppliers, and may suffer from security and privacy issues (D7). Still, it is worth noting the relationship with public DLTs.

### 5.3. Distributed Ledger Technology

When a WSDB employs the DLT, an important decision is related to the most suitable type of DLT to be used. The range of options includes blockchain, Tangle or other hash-based graphs for information registry and processing (D1–D3, D8–D9, D11–D12). Specifically, with respect to blockchain, two important question are: (i) how the blocks are closed? (ii) how the consensus is obtained among nodes to validate closed blocks. The typical approach is proof-of-work (PoW). Alternatively, proof-of-stake (PoS) and distributed PoS (dPoS) are emerging. Consensus techniques are also evolving. Novel approaches derived from the practical byzantine fault tolerance (PBFT) algorithm are being employed [17]. Tangle does not close blocks. A novel transaction validates two random selected previous ones—an approach that appears to be more scalable (D5) and efficient (D3 and D4) than blockchain. In summary, some important questions are: (i) which of these technologies are better for a dynamic spectrum market (mainly D4–D7, D11)? (ii) which has the best performance and transaction costs (D3 and D10)?

### 5.4. The Role of DLT in Architecture

Are DLTs aimed at storing all control data (D1)? Or are they responsible only for storing secondary spectrum allocations? Or should they be employed for full monetization of the spectrum market (D11)? Assessing DLTs scope in architecture is critical. Although DLTs offer guarantees not available in traditional databases, this comes at a price (D4 and D5). Investigating the various possibilities and evaluating the performance of each one is an important subject for future work.

### 5.5. Distributed Ledger Centralization Versus Decentralization

DLTs can be centralized or decentralized regarding nodes topology. The centralized DLT has a unique authority to handle the ledger, whereas the decentralized one have network nodes with the ability of maintaining an independent shared ledger (D5). At a first glance, the centralized blockchain seems to be vulnerable to the single point of failure problem, while the decentralized has a natural defense against this problem (D7). Also related to this issue, how much nodes should participate in a DLT network to provide guarantees of information immutability is of concern. Changing block history on a large number of nodes simultaneously (during a block time) appears to be more difficult than on 2 or 3 nodes.

### 5.6. Public Versus Private Blockchain

A public or open DLT allows anyone to join with full participation in the network, while a private blockchain has restrictions on who can participate. The decision upon which one to adopt is related to dimensions D3–D7, D10–D11. Once the framework under consideration concerns to a very specific set of network operators, the private blockchain could be more suitable to this group of users, forming a consortia for DSA market. However, the evaluation of pros and cons is only at the beginning.

### 5.7. Distributed Ledger Energy Expenditure and Scalability

A proof-of-work blockchain may have scalability limitations and high energy expenditure (D6) at nodes. A blockchain using other approaches has smaller energy fingerprint, but may have implications regarding information reliability and immutability (D3). On the other hand, Tangle still uses third-party networks to carry out transaction validations, being therefore not fully decentralized. Modernizing the architecture as DLTs evolve seems to be a demand that is here to stay.

### 5.8. Smart Contracts

To use or not smart contracts is a difficult decision, which affects D3, D7, D10 and D11, mainly. For instance, Ethereum takes advantages of the smart contracts, while IOTA’s Tangle does not fully implement smart contracts yet. However, IOTA proof-of-work appears to be faster (D4) and more scalable (D5) than Ethereum. Assessing whether immutable computing is an important demand or not help untie this difficulty.

### 5.9. Data, Services and Database/DLT Interoperability

Interoperability (D13) is a problem that needs to be addressed, from the data coming out of the spectrum sensors to the storage in a traditional database or DLT. The interoperability of architectural services is also an important concern.

## 6. Conclusions

This Review discussed about a DLT-enabled DSA framework in which a WSDB with high confidentiality, integrity, availability, and authenticity is fed by spectrum sensing events, taking advantage of a supporting IoT network equipped with spectrum sensors, the SSIoTs. The high enough node density and dedicated sensors could provide a fine-grid real-time spectrum occupancy map for diverse wireless applications. It is envisaged that only the WSDB information will be eventually used to support DSA. Thus, the hardware complexity of the SUs is not increased, since the spectrum sensing task is shifted from the secondary network to the supporting IoT network. Moreover, the intrinsic inaccuracy of propagation prediction models is avoided, since the spectrum availability data is not formed from such a source of spectrum occupancy.

When a WSDB employs the DLT, an important decision concerns the most suitable type of DLT to be used. The range of options includes blockchains with proof-of-work or other consensus mechanisms, Tangle or other hash-based graphs for information registry and processing. DLTs can be also centralized or decentralized regarding nodes topology, public or private with respect to how stakeholders access the database. The centralized DLT has a central authority to handle the ledger, whereas the decentralized one have network nodes with the ability of maintaining an independent shared ledger. At a first glance, the centralized blockchain seems to be vulnerable to the single point of failure problem, while the decentralized has a natural defense against this limitation. A public or open DLT allows anyone to join with full participation in the network, while a private blockchain has restrictions on who can participate. Once the framework concerns to a very specific set of network operators, the private blockchain would be more suitable to these users, forming a consortia for DSA market.

A blockchain with proof-of-work may have scalability limitations and high energy expenditure at nodes. A blockchain using other approaches has smaller energy fingerprint, but may have implications regarding information reliability and immutability. On the other hand, Tangle still uses third-party networks to carry out transaction validations, being therefore not fully decentralized.

Another aspect of concern is the use or not of smart contracts. For instance, Ethereum takes advantages of the smart contracts, while IOTA’s Tangle does not fully implement smart contracts yet. However, IOTA proof-of-work appears to be faster and more scalable than Ethereum.

Finally, there is the issue of the role of the DLT in the architecture: Is it aimed at storing all data and transactions related to spectrum market, at storing only secondary spectrum allocation decisions, or just for monetization of the spectrum market? The optimal approach is a matter of future work.

We envisage a complete ’things and spectrum market’ that can be built by integrating SSIoTs with DLTs and other databases running in cloud computing, with primary and secondary networks dynamically negotiating opportunities, performing control and management functionalities via smart contracts.

## Figures and Tables

**Table 1:** Design dimensions considered in this Review.

Dimension	Description
D1	Dynamic spectrum sharing and access policies.
D2	Spectrum sensing.
D3	Reliability, efficiency and consistency of spectrum information sharing and opportunistic decisions.
D4	Realtimeness.
D5	Low complexity and scalability.
D6	Energy fingerprint.
D7	Confidentiality, integrity, availability, authenticity, and immutability.
D8	Spectrum sensing coverage.
D9	Location awareness.
D10	Cost-effectiveness.
D11	Spectrum market as a service.
D12	Artificial intelligence and machine learning.
D13	Equipment, data, services and platforms interoperability.

**Table 2:** Related work on DSA. Scope comparison with respect to: D1—Dynamic spectrum sharing and access policies; D2—Spectrum sensing; D3—Reliability, efficiency and consistency of spectrum information sharing and opportunistic decisions; D4—Realtimeness; D5—Low complexity and scalability; D6—Energy fingerprint; D7—Security and immutability; D8—Spectrum sensing coverage; D9—Location awareness; D10—Cost-effectiveness; D11—Spectrum market as a service; D12—AI and ML; D13—Interoperability.

	Dimensions												
**Related Work**	**D1**	**D2**	**D3**	**D4**	**D5**	**D6**	**D7**	**D8**	**D9**	**D10**	**D11**	**D12**	**D13**
Tang et al. [38]		✓	✓					✓	✓				
Aslam et al. [39]		✓	✓						✓	✓			✓
FCC [27,28]	✓	✓	✓				✓	✓	✓		✓		✓
Vartiainen et al. [40]	✓	✓						✓	✓				✓
Paisana et al. [37]		✓	✓	✓				✓	✓				
Dionisio et al. [41]		✓	✓	✓	✓			✓	✓	✓			✓
Tran et al. [43]	✓	✓		✓	✓	✓		✓	✓				
Qin et al. [13]		✓		✓	✓	✓		✓	✓				
Zhang et al. [44]		✓		✓	✓	✓		✓	✓	✓			
Wang et al. [35]		✓	✓	✓				✓	✓				
Ma et al. [45]		✓		✓	✓	✓		✓	✓	✓			
Ariyarathna et al. [14]	✓						✓				✓		
Weiss et al. [46]	✓		✓				✓				✓		
Chen et al. [47]			✓				✓				✓		
Hartog et al. [48]			✓				✓						
Bayhan et al. [50]	✓	✓	✓				✓	✓		✓	✓		✓
Maksymyuk et al. [51]	✓		✓	✓	✓		✓			✓	✓	✓	✓
Liu et al. [52]				✓	✓	✓	✓			✓		✓	✓
Ali et al. [53]				✓	✓		✓			✓			✓

## References

[1] Hong X., Wang J., Wang C.X., Shi J. (2014). Cognitive radio in 5G: A perspective on energy-spectral efficiency trade-off. IEEE Commun. Mag..

[2] Zhang L., Xiao M., Wu G., Alam M., Liang Y.C., Li S. (2017). A survey of advanced techniques for spectrum sharing in 5G networks. IEEE Wirel. Commun..

[3] Mitola J., Maguire G.Q. (1999). Cognitive radio: Making software radios more personal. Pers. Commun. IEEE.

[4] Goldsmith A., Jafar S.A., Maric I., Srinivasa S. (2009). Breaking Spectrum Gridlock With Cognitive Radios: An Information Theoretic Perspective. Proc. IEEE.

[5] Corral-de-Witt D., Ahmed S., Awin F., Rojo-Álvarez J.L. (2019). An Accurate Probabilistic Model for TVWS Identification. Appl. Sci..

[6] Holland O., Bogucka H., Medeisis A. (2015). Opportunistic Spectrum Sharing and White Space Access: The Practical Reality.

[7] Harada H. (2014). White space communication systems: An overview of regulation, standardization and trial. IEICE Trans. Commun..

[8] IEEE (2011). IEEE Standard for Information Technology—Local and Metropolitan Area Networks—Specific Requirements—Part 22: Cognitive Wireless RAN Medium Access Control (MAC) and Physical Layer (PHY) Specifications: Policies and Procedures for Operation in the TV Bands.

[9] Federal Communications Commission (2014). Amendment of Part 15 of the Commission’s Rules for Unlicensed Operations in the Television Bands, Repurposed 600 MHz Band, 600 MHz Guard Bands and Duplex Gap, and Channel 37: Notice of Proposed Rulemaking.

[10] Ofcom A Framework for Spectrum Sharing. https://www.ofcom.org.uk/__data/assets/pdf_file/0032/79385/spectrum-sharing-framework.pdf.

[11] Akyildiz I.F., Lo B.F., Balakrishnan R. (2011). Cooperative spectrum sensing in cognitive radio networks: A survey. Phys. Commun..

[12] Arjoune Y., Kaabouch N. (2019). A comprehensive survey on spectrum sensing in cognitive radio networks: Recent advances, new challenges, and future research directions. Sensors.

[13] Qin Z., Gao Y., Parini C.G. (2016). Data-Assisted Low Complexity Compressive Spectrum Sensing on Real-Time Signals under Sub-Nyquist Rate. IEEE Trans. Wirel. Commun..

[14] Ariyarathna T., Harankahadeniya P., Isthikar S., Pathirana N., Bandara H.M.N.D., Madanayake A. Dynamic Spectrum Access via Smart Contracts on Blockchain. Proceedings of the 2019 IEEE Wireless Communications and Networking Conference (WCNC).

[15] Danzi P., Kalor A.E., Sorensen R.B., Hagelskjaer A.K., Nguyen L.D., Stefanovic C., Popovski P. (2020). Communication Aspects of the Integration of Wireless IoT Devices with Distributed Ledger Technology. IEEE Netw..

[16] Belotti M., Božić N., Pujolle G., Secci S. (2019). A Vademecum on Blockchain Technologies: When, Which, and How. IEEE Commun. Surv. Tutor..

[17] Abbade L.R., Ribeiro F.M., d. Silva M.H., Morais A.F.P., d. Morais E.S., Lopes E.M., Alberti A.M., Rodrigues J.J.P.C. (2020). Blockchain Applied to Vehicular Odometers. IEEE Netw..

[18] Bozic N., Pujolle G., Secci S. Securing virtual machine orchestration with blockchains. Proceedings of the 2017 1st Cyber Security in Networking Conference (CSNet).

[19] Tasic A.M., Narathong C., Sahota G.S., Patel S. (2015). Spectrum Sensing Radio Receiver. Patent.

[20] Ramirez D., Via J., Santamaria I., Scharf L.L. (2013). Locally Most Powerful Invariant Tests for Correlation and Sphericity of Gaussian Vectors. IEEE Trans. Inf. Theory.

[21] Chaurasiya R.B., Shrestha R. (2020). Area-Efficient and Scalable Data-Fusion based Cooperative Spectrum Sensor for Cognitive Radio. IEEE Trans. Circuits Syst. II Express Briefs.

[22] Guimarães D.A. (2020). Pietra-Ricci Index Detector for Centralized Data Fusion Cooperative Spectrum Sensing. IEEE Trans. Veh. Technol..

[23] IEEE (2014). IEEE Standard for Information Technology—Telecommunications and Information Exchange between Systems—Local and Metropolitan Area Networks—Specific Requirements—Part 11: Wireless LAN Medium Access Control (MAC) and Physical Layer (PHY) Specifications Amendment 5: Television White Spaces (TVWS) Operation.

[24] Zhu L., Chen V., Malyar J., Das S., McCann P. (2015). Protocol to Access White-Space (PAWS) Databases. Internet Engineering Task Force.

[25] Luo Y., Gao L., Huang J. (2016). Economics of Database-Assisted Spectrum Sharing.

[26] Federal Communications Commission (2015). Amendment of Part 15 of the Commission’s Rules for Unlicensed Operations in the Television Bands, Repurposed 600 MHz Band, 600 MHz Guard Bands and Duplex Gap, and Channel 37.

[27] Federal Communications Commission (2020). Wireless Telecommunications Bureau and Office of Engineering and Technology Approve Four Spectrum Access System Administrators for Full Scale Commercial Deployment in the 3.5 GHz Band and Emphasize Licensee Compliance Obligations in the 3650–3700 MHz Band under Part 96.

[28] Federal Communications Commission 3.5 GHz Band Overview. https://www.fcc.gov/wireless/bureau-divisions/mobility-division/35-ghz-band/35-ghz-band-overview.

[29] LS Telcom Channel Availability. https://www.whitespaceforus.com_wsdb_wsdb_ui_Channel_Availability.html.

[30] Spiridon S. (2016). Toward 5G Software Defined Radio Receiver Front-Ends.

[31] Shairi N.A., Ahmad B., Wong P. SPDT discrete switch design using switchable radial stub resonator for WiMAX and LTE in 3.5 GHz band. Proceedings of the 2013 IEEE International RF and Microwave Conference (RFM).

[32] Shellhammer S.J. Spectrum sensing in IEEE 802.22. Proceedings of the IAPR Workshop on Cognitive Information Processing (CIP 2008).

[33] Ma Y., Zhang X., Gao Y. (2017). Joint sub-Nyquist spectrum sensing scheme with geolocation database over TV white space. IEEE Trans. Veh. Technol..

[34] Ofcom The Wireless Telegraphy (White Space Devices) (Exemption) Regulations 2015. https://www.legislation.gov.uk/uksi/2015/2066/pdfs/uksi_20152066_en.pdf.

[35] Wang N., Gao Y., Evans B. Database-augmented spectrum sensing algorithm for cognitive radio. Proceedings of the 2015 IEEE International Conference on Communications (ICC).

[36] Stevenson C.R., Chouinard G., Lei Z., Hu W., Shellhammer S.J., Caldwell W. (2009). IEEE 802.22: The first cognitive radio wireless regional area network standard. IEEE Commun. Mag..

[37] Paisana F., Miranda J.P., Marchetti N., Dasilva L.A. Database-aided sensing for radar bands. Proceedings of the 2014 IEEE International Symposium on Dynamic Spectrum Access Networks, DYSPAN 2014.

[38] Tang M., Zheng Z., Ding G., Xue Z. Efficient TV white space database construction via spectrum sensing and spatial inference. Proceedings of the 2015 IEEE 34th International Performance Computing and Communications Conference (IPCCC).

[39] Aslam S., ul Haq A., Jang J.W., Lee K.G. (2018). Unified channel management for cognitive radio sensor networks aided internet of things. Sensors.

[40] Vartiainen J., Matinmikko-Blue M., Karvonen H., Mendes L. Spectrum Sharing and Operator Model for Rural and Remote Area Networks. Proceedings of the 2019 16th International Symposium on Wireless Communication Systems (ISWCS).

[41] Dionísio R., Ribeiro J., Marques P., Rodriguez J. (2014). Combination of a geolocation database access with infrastructure sensing in TV bands. Eurasip J. Wirel. Commun. Netw..

[42] Cognitive Radio Experimentation World Project Overview|CREW Project. http://www.crew-project.eu/index.html.

[43] Tran H.N., Sun C., Alemseged Y.D., Harada H. (2012). A distributed sensing and caching database for cognitive radio systems. IEICE Trans. Commun..

[44] Zhang X., Ma Y., Qi H., Gao Y., Xie Z., Xie Z., Zhang M., Wang X., Wei G., Li Z. (2018). Distributed Compressive Sensing Augmented Wideband Spectrum Sharing for Cognitive IoT. IEEE Internet Things J..

[45] Ma Y., Zhang X., Gao Y. An Efficient Joint Sub-Nyquist Spectrum Sensing Scheme with Geolocation Database over TV White Space. Proceedings of the 2017 IEEE International Conference on Communications (ICC).

[46] Weiss M.B.H., Werbach K., Sicker D.C., Bastidas C.E.C. (2019). On the Application of Blockchains to Spectrum Management. IEEE Trans. Cogn. Commun. Netw..

[47] Chen Y., Gu J., Chen S., Huang S., Wang X.S. A Full-Spectrum Blockchain-as-a-Service for Business Collaboration. Proceedings of the 2019 IEEE International Conference on Web Services (ICWS).

[48] d. Hartog F., Bouhafs F., Shi Q. Toward secure trading of unlicensed spectrum in cyber-physical systems. Proceedings of the 2019 16th IEEE Annual Consumer Communications Networking Conference (CCNC).

[49] Yuan P., Xiong X., Lei L., Zheng K. (2019). Design and Implementation on Hyperledger-Based Emission Trading System. IEEE Access.

[50] Bayhan S., Zubow A., Wolisz A. Smart contracts for spectrum sensing as a service. Proceedings of the 2018 IEEE International Symposium on Dynamic Spectrum Access Networks (DySPAN).

[51] Maksymyuk T., Gazda J., Volosin M., Bugar G., Horvath D., Klymash M., Dohler M. (2020). Blockchain-Empowered Framework for Decentralized Network Management in 6G. IEEE Commun. Mag..

[52] Liu Y., Yu F.R., Li X., Ji H., Leung V.C.M. (2020). Blockchain and Machine Learning for Communications and Networking Systems. IEEE Commun. Surv. Tutor..

[53] Ali M.S., Vecchio M., Pincheira M., Dolui K., Antonelli F., Rehmani M.H. (2019). Applications of Blockchains in the Internet of Things: A Comprehensive Survey. IEEE Commun. Surv. Tutor..

[54] Leens F. (2009). An introduction to I^2^C and SPI protocols. Instrum. Meas. Mag. IEEE.

[55] Wardana A.A., Perdana R.S. Access Control on Internet of Things based on Publish/Subscribe using Authentication Server and Secure Protocol. Proceedings of the 2018 10th International Conference on Information Technology and Electrical Engineering (ICITEE).

[56] Ofcom Qualifying White Space Databases. https://tvws-databases.ofcom.org.uk/.

[57] Akyildiz I.F., Su W., Sankarasubramaniam Y., Cayirci E. (2002). A survey on sensor networks. IEEE Commun. Mag..

[58] Nakamoto S. Bitcoin: A Peer-to-Peer Electronic Cash System. https://bitcoin.org/bitcoin.pdf.

[59] Pinna A., Ibba S., Baralla G., Tonelli R., Marchesi M. (2019). A Massive Analysis of Ethereum Smart Contracts Empirical Study and Code Metrics. IEEE Access.

[60] Korotkyi I., Sachov S. Hardware Accelerators for IOTA Cryptocurrency. Proceedings of the 2019 IEEE 39th International Conference on Electronics and Nanotechnology (ELNANO).

[61] Bayhan S., Zubow A., Wolisz A. Spass: Spectrum Sensing as a Service via Smart Contracts. Proceedings of the 2018 IEEE International Symposium on Dynamic Spectrum Access Networks, DySPAN 2018.

[62] Kotobi K., Bilen S.G. (2018). Secure Blockchains for Dynamic Spectrum Access: A Decentralized Database in Moving Cognitive Radio Networks Enhances Security and User Access. IEEE Veh. Technol. Mag..

[63] Popov S. The Tangle. https://www.iota.org/foundation/research-papers.

[64] Silvano W.F., Marcelino R. (2020). Iota Tangle: A cryptocurrency to communicate Internet-of-Things data. Future Gener. Comput. Syst..

[65] Holl P., Scepankova E., Matthes F. (2018). Smart Contract based API usage tracking on the Ethereum Blockchain. Software Engineering und Software Management 2018.

[66] IOTA Foundation The State of Qubic and the future of Smart Contracts on IOTA. https://blog.iota.org/the-state-of-qubic-63ffb097da3f.

[67] Lamtzidis O., Pettas D., Gialelis J. (2019). A Novel Combination of Distributed Ledger Technologies on Internet of Things: Use Case on Precision Agriculture. Appl. Syst. Innov..

[68] Živi N., Kadušić E., Kadušić K. Directed Acyclic Graph as Tangle: An IoT Alternative to Blockchains. Proceedings of the 2019 27th Telecommunications Forum (TELFOR).

[69] Tzianos P., Pipelidis G., Tsiamitros N. Hermes: An Open and Transparent Marketplace for IoT Sensor Data over Distributed Ledgers. Proceedings of the 2019 IEEE International Conference on Blockchain and Cryptocurrency (ICBC).

[70] Ferraro P., King C., Shorten R. (2018). Distributed Ledger Technology for Smart Cities, the Sharing Economy, and Social Compliance. IEEE Access.

